# Associations Between Distinct Trauma Classes and Mental Health Care Utilization in Norwegian Adolescents: A National Registry Study

**DOI:** 10.1007/s10578-024-01671-9

**Published:** 2024-02-08

**Authors:** Annika Skandsen, Sondre Aasen Nilsen, Mari Hysing, Martin H. Teicher, Liv Sand, Tormod Bøe

**Affiliations:** 1 Department of Psychosocial Science, Faculty of Psychology, University of Bergen, Bergen, Norway; 2 Stavanger University Hospital, Stavanger, Norway; 3 Regional Centre for Child and Youth Mental Health and Child Welfare, NORCE Norwegian Research Centre, Bergen, Norway; 4 Department of Psychiatry, Harvard Medical School, Boston, MA USA; 5 Developmental Biopsychiatry Research Program, McLean Hospital, Belmont, MA USA

**Keywords:** Potentially traumatic life experiences, Latent class analysis, Diagnosis, Adolescence, CAMHS

## Abstract

Adolescents who experience potentially traumatic experiences (PTEs) have an increased risk of psychopathology. PTEs often co-occur and may form interrelated patterns of exposure. This study investigated underlying classes of PTE exposure among Norwegian adolescent participants in the youth@hordaland study, and whether such classes were associated with contact with child and adolescent mental health services (CAMHS) and psychiatric diagnoses. The data stem from the population-based youth@hordaland study conducted in 2012 which was linked to the Norwegian Patient Registry (NPR, *n* = 8845). Exposure to PTEs was assessed by adolescent self-report whereas psychiatric disorders (Axis 1) were derived from the NPR. Latent Class Analysis was used to identify distinct classes of PTE exposure-patterns in the data. Logistic regression analyses were performed to investigate associations between classes of PTEs and contact with CAMHS and psychiatric diagnoses. Three classes of PTE exposure were identified based on model fit indices and theoretical considerations. Compared with participants in the low trauma class (88% of participants), those in the Situational-(6%) and Interpersonal trauma class (6%) had higher odds-ratios (ORs) for contact with CAMHS (OR = 2.27 (95% CI [1.78, 2.87])) and (OR = 3.26 (95% CI [2.61, 4.04])) respectively, and for being diagnosed with a psychiatric disorder in CAMHS (ORs ranged from 2.19 – 10.4) after adjusting for sex and parental education. There were more participants diagnosed with ADHD within the Interpersonal trauma class compared to the Situational trauma class when adjusting for sex and parental education (OR = 2.22 (95% CI [1.17, 4.40])). Three relatively homogeneous PTE classes, consisting of distinct patterns of trauma exposure were associated with a higher odds of contact with CAMHS and of being diagnosed with a psychiatric disorder in CAMHS. The study highlights the co-occurrence of PTEs and their impact across the diagnostic spectrum.

## Introduction

Potentially traumatic experiences (PTEs) can be defined as exposure to an event involving threat, actual or perceived, to the life or physical safety of the individual, their loved ones or those around them [36] and may have a broad impact on mental health [19, 43, 44]

Research has increasingly showed that PTEs often co-occur [14, 35] which point towards the possibility that PTEs might represent an interrelated pattern of traumatic experiences [35].

Factor analysis studies have shown that traumatic events might cluster in predictable ways [4, 41]. However, such a PTE centered approach entails the assumption that a population is homogenous in terms of how variables influence one another and subsequent outcome. Different groups of individuals may differ in their patterns of trauma exposure and consequently constitute a more heterogenous group than factor analysis studies might contemplate. Hence, by using a person-centered approach when examining clusters of PTEs one may identify homogenous subgroups while recognizing the heterogeneity of PTEs experienced at a population level.

A systematic review of person-centered approaches by [35] found that individuals grouped together across PTEs and formed reliable classes. Most of the studies measured Interpersonal trauma and identified a subgroup that was characterized by higher likelihood of *all* Interpersonal traumas. Studies that measured both Interpersonal and non-Interpersonal trauma, identified a group with a higher probability of being exposed to all types of PTEs. The largest number of participants were characterized by low risk of exposure to any PTE.

A US cross-sectional study of adolescents (N = 5870) identified three latent classes of PTEs in adolescents: physical neglect/emotional abuse/household dysfunction; physical abuse/emotional abuse/household dysfunction; and emotional abuse/caregiver divorce [6]. However, the study only included adolescents in families investigated for maltreatment. Hence, the identified PTE sub-groups may not generalize to the wider adolescent population. Indeed, in another representative US study of general population youth, four distinct trauma classes were identified: high risk, sexual assault risk, exposure to non-sexual trauma, and low risk [30, 35]

Few studies have examined how trauma classes are related to psychiatric disorder, although a few notable exceptions exist. The study by McChesney et al. [30] found that the likelihood of mood and anxiety disorders were significantly higher in the three trauma classes compared to the low risk class. Moreover, the trauma classes had a significantly higher probability of PTSD when compared to the low-risk class, with those in the sexual assault class being over 26 times as likely to have PTSD.

A retrospective national US study revealed a 3-class model of childhood trauma in which 85% of the participants were allocated to a low trauma class, 6% to a multi-victimization class (reporting exposure to all types of relational PTEs) and 9% to a Situational trauma-class (exposed to a range of PTEs) [15]. Both trauma groups showed increased psychopathology when assessed with a personal computer-assisted diagnostic interview, measuring mood disorders, anxiety disorders, PTSD, personality disorders and substance use disorders. Rates of PTSD were elevated in both childhood trauma profiles, the multi-victimization class (*β* = 0.46) and the Situational trauma-class (*β* = 0.33).

When studying the association between subgroups of PTEs and psychopathology, accounting for sex and socioeconomic status (SES) is important, as both are associated with PTEs and psychiatric diagnoses [34, 48]. For example, girls tend to more often be in a high exposure subgroup of PTEs, and to experience sexual trauma [2, 21]; [30]. Boys, on the other hand, have a higher likelihood of being in a loss/violence subgroup [2]. It is also documented that the prevalence of various psychiatric disorders differs between boys and girls [16, 30]. Moreover, children from low-SES families are disproportionately exposed to more adverse physical and social environmental conditions and have more psychiatric disorders than more affluent peers [7, 25].

Based on the above considerations, this study sought to investigate patterns of PTE exposure among a general population of youths from Norway and whether patterns of PTE exposure were associated with CAMHS contact and psychiatric disorders. This study contributes to the field of trauma by building on the limited research into PTE classes and psychopathology in adolescence, by using large-scale data on a well-defined cohort of older adolescents that were linked to patient registry data. To our knowledge, no studies have examined these associations across the diagnostic spectrum by using national patient registry data.

## Method

### Procedure

This study uses data from a Norwegian population-based study of adolescents in Hordaland County, the youth@hordaland-survey [24, 42]. The main aim of the survey was to assess the prevalence of mental health problems and service use in adolescents. During the first month of 2012, all adolescents born between 1993 and 1995 were invited to participate in the epidemiological study (N = 19 439). Participants in upper secondary education received study information via school e-mail while information was sent by postal mail to adolescents not in school. The questionnaire was web-based, and one school hour was assigned to complete the questionnaire. The questionnaire covered demographic characteristics, daily life functioning, use of health care and social services and a broad range of mental health issues. Data from the youth@hordaland study was linked to the National Patient Registry (NPR) for those who consented to register linkage. NPR is the official registry for specialist health care in Norway and provide data from CAMHS. The registry owner at the Norwegian Directorate of Health conducted the linkage.

### Participants

A total of 10 257 adolescents aged 16–19 years agreed to participate in the survey (participation rate: 53%) of which 9555 consented to registry linkage. Participants were removed from the sample if they had missing information on all the variables assessing PTEs, and if they reported being older when the event happened than their actual age at the time of participation. The final sample size for the present study was 8755 (85%). Out of the final sample, 905 (10.3%) had at least one registration in NPR. A previous study on the linkage between NPR and youth@hordaland found that participants who did not consent to the linkage were somewhat older, had higher mean number of self-reported conduct problems and had somewhat higher frequencies of high-level alcohol consumption. Nevertheless, the differences were small, with Cohen’s ds ranging from 0.09 to 0.26 [20]. In the present study, there was no significant difference in mean number of PTE exposure between participants in the youth@hordaland survey who consented to registry linkage compared to those who did not consent (*M*_difference_ = 0.02), *p* = 0.335, *d* = -0.024.

### Measures

#### Socio-Demographics

Date of birth and sex were obtained from the personal identity number in the Norwegian National Population Register. Paternal and maternal education were reported separately by adolescents and categorized into “primary school”, “secondary school” and “college or university”.

#### Potentially Traumatizing Experiences (PTEs)

We used self-report data from the youth@hordaland-survey to obtain information on adolescent PTE exposure. The items chosen to measure PTEs was based on the HUNT-3 negative life event checklist, included in a Norwegian population-based study [26] and has been used in other youth@hordaland studies [7, 44].

A total of ten PTEs were measured. The ten PTEs were derived from five items: had the adolescents ever experienced: “a catastrophe or serious accident”, “violence from a grown-up”, “witnessed someone you care about being exposed to violence from a grown-up”, “unwanted sexual actions” and “death of someone close to you”. If the adolescent had experienced death of someone close, they were asked to specify who that person was with the alternatives: “death of a parent/guardian”, “death of a sibling”, “death of a grandparent”, “death of another family member close to you”, “death of a close friend”, or “death of a boyfriend/girlfriend”. Multiple responses were possible. The response alternatives were either “no, never”, “yes, once”, “yes, sometimes” and “yes, several times” or for some items “no, never”, “yes, once” and “yes, more than once”. All responses, except “no, never”, were collapsed to indicate PTE exposure. We did not have information on the timing of the PTE exposure in relation to CAMHS contact, other than 8.8% of the participants having been in contact with CAMHS after the youth@hordaland survey, which indicated PTE exposure prior to mental health problems. The item related to the death of a loved one did not assess whether the death was expected or sudden, as the death of nearly anyone close to an adolescent, apart, perhaps, from a grandparent, could be considered a PTE. Death of a grandparent was accordingly omitted from the analysis. Our items aligned with the Child and Adolescent Trauma Screen 2 (CATS-2; [38], but did not have the same level of detail as this checklist. CATS- 2 assesses PTEs using a 15-item structured PTE checklist. As opposed to CATS- 2 our checklist did not include including questions covering community violence, bullying, war and medical trauma.

#### Psychiatric Disorders and Referrals

Psychiatric disorders were obtained from the Axis 1 clinical diagnoses from the Norwegian Patient Registry (NPR), ascertained using criteria in the *International Classification of Diseases and Disorders* (10th ed.; WHO, 2016), coded by clinicians working in CAMHS. A total of 133 adolescents had psychiatric diagnoses from more than one of the specified diagnostic categories and were assigned to multiple diagnostic categories, whereas 329 participants did not receive any Axis 1 psychiatric diagnosis [20]. The latter group was in contact with CAMHS, but their mental health concerns were not sufficient to fulfill the criteria for any Axis 1 psychiatric diagnosis; hence, they were excluded from the analyses on register-based diagnoses. The data from the NPR spanned from January 2008 to March 2018. Participants participated in the youth@hordaland at the age of 16–19, and their data obtained from CAMHS spanned from their age of 12 to 19 years. We assigned all the diagnoses into broader diagnostic categories: anxiety disorders; mood disorders; trauma-related disorders; eating disorders; autism spectrum disorders (ASD); conduct disorders; attention-deficit/hyperactivity disorder (ADHD); and psychotic disorders [20]. Due to the small group sizes of the diagnostic groups Autism, Psychosis and Eating disorders were removed from further analysis.

### Ethics

The Regional Committee for Medical and Health Research Ethics (REC) in Western Norway (2012/1467/REK Vest) and SIKT (Norwegian Agency for Shared Services in Education and Research; 371 974 and 259 631) approved the study. Following the regulations from the Norwegian health authorities and REC, adolescents aged 16 years and older can make decisions regarding their health (including participation in health studies), and thus provided written consent to participate in the study and for the linkage to registries. Parents or guardians of participants younger than 18 years of age received information about the study but did not decide if the teenager could participate.

### Statistical Analysis

We used latent class analysis and tested solutions from two to eight classes for the 10 PTE variables. To decide on the number of classes in the final model, we relied on entropy [49] and the following information criteria: Bayesian Information Criterion (BIC; [39], Akaike Information Criterion (AIC; [3], and Consistent Akaike Criterion (cAIC; [3]. BIC metric is often considered the most reliable fit statistic in LCA [52]. The fit indices were compared for the different solutions to determine the number of classes in combination with theoretical considerations and the interpretability of the solution. The optimal class solution had the lowest BIC value, relatively higher entropy values, and had to give conceptual and interpretive meaning.

Following the determination of latent classes, descriptive analyses were performed to compare sociodemographic characteristics across classes. Next, we used logistic regression analyses to examine 1) whether class membership was related to contact with CAMHS, and 2) whether class membership was associated with psychiatric diagnoses. These analyses were conducted in three steps: a) a crude model without any adjustments, b) a model adjusting for sex, and c) a model adjusting for sex and maternal-and paternal education. Class 1 (the most prevalent class) was used as a reference group in the analyses, but we also conducted pairwise tests between the other classes. To ease the dissemination of the results, we also present the main regression results visually as a regression coefficient plot, where the associations between the latent classes and psychiatric diagnosis are sorted based on the magnitude of the associations. There were few missing values for the covariates included in the analyses (all < 1%). Missing data was handled by pairwise-deletion in the regression models.

All analysis were performed in R statistical software [37], using the package *poLCA* [29] and ggplot2. [51]

## Results

### Latent PTE Classes and Characteristics of the Sample

Table 1 presents the LCA results for different class models. As shown in Table 1, the information criteria and entropy were most supportive of a three-class model. That is, the BIC and cAIC were lowest for the three-class solution. AIC, on the other hand, indicated that an eight-class model had the best fit. However, as we placed more weight to the BIC metric, we selected the three-class model. The three-class model also appeared the most justified based on previous LCA studies (i.e. [30, 35] and categorization of PTEs within the field of trauma [5]; [15]. Based on the frequency and distributions of PTEs in the three classes, we labelled the classes Low trauma class, Situational trauma class, and Interpersonal trauma class.

**Table 1:** Latent class analysis: Model fit statistics for two through to eight PTE classes

N.class	AIC	cAIC	BIC	Entropy
2	56,339.27	56,509.07	56,488.07	0.64
**3**	**56,229.16**	**56,487.91**	**56,455.91**	**0.72**
4	56,174.91	56,522.61	56,479.61	0.67
5	56,158.32	56,594.96	56,540.96	0.63
6	56,147.93	56,673.53	56,608.53	0.76
7	56,126.48	56,741.02	56,665.02	0.71
8	56,126.30	56,829.78	56,742.78	0.73

Bold = class model chosen

PTE = Potentially traumatic experiences, AIC = Akaike Information Criterion, cAIC = Consistent Akaike Criterion, BIC = Bayesian information Criterion

**Fig. 1:**
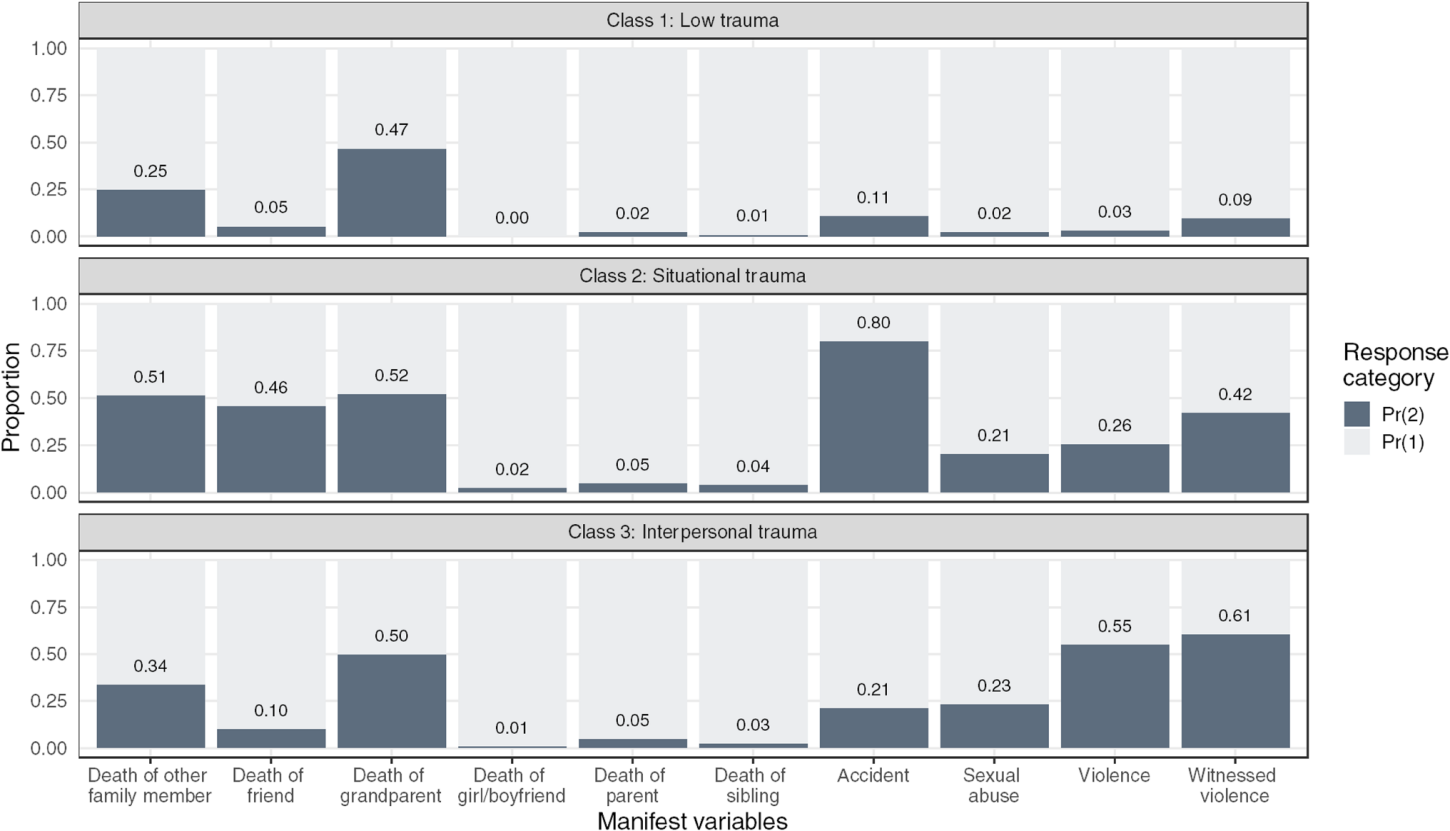
Description of Classes of Potentially Traumatic Experiences (PTEs) based on manifest variables. This figure shows the proportion of all individual potentially traumatic experiences (PTEs; manifest variables) by latent classes. Pr(2): Exposure to PTE, Pr(1): No exposure to PT

Most adolescents were in the Low trauma class (88% of participants; class 1 in Fig. 1). This class had low exposure to most PTEs, except death of a grandparent and other family member. The Situational trauma class*,* consisted of 6% of the sample and was characterized by a high proportion having experienced accidents and multiple deaths (including of close friends), and adolescents in this class also had some exposure to Interpersonal PTEs (i.e., sexual abuse, violence and witnessed violence). The Interpersonal trauma class consisted of 6% of the sample who had experienced a higher frequency of violence and who had witnessed violence, and who also had some exposure to sexual abuse, accidents, and deaths within the family (see Fig. 1).

Descriptively, there were no age differences between the classes. Sex was distributed equally in the low trauma class, but there were more girls than boys in the Situational trauma class (66% girls) and in the Interpersonal trauma class (70% girls). More adolescents perceived their economic well-being as poorer in the Situational-(11%) and Interpersonal (17%) trauma classes compared to adolescents within the Low trauma group (6.1%). Fewer parents of youths in the two trauma classes had higher levels of education (see Table 2 for details).

**Table 2:** Demographic characteristics across PTE classes

Characteristic	Low trauma class N = 7,701^1^ (88%)	Situational trauma class N = 525^1^ (6%)	Interpersonal trauma class, N = 529^1^ (6%)
Sex			
Female	4,006 (52%)	345 (66%)	370 (70%)
Male	3,695 (48%)	180 (34%)	159 (30%)
Maternal education			
Elementary	566 (7.4%)	47 (9.0%)	59 (11%)
Intermediate	2,415 (32%)	177 (34%)	173 (33%)
Higher	2,884 (38%)	185 (36%)	180 (34%)
Unknown	1,790 (23%)	112 (21%)	113 (22%)
Missing	46	4	4
Paternal education			
Elementary	601 (7.9%)	44 (8.5%)	50 (9.6%)
Intermediate	2,652 (35%)	198 (38%)	177 (34%)
Higher	2,518 (33%)	143 (28%)	144 (28%)
Unknown	1,875 (25%)	132 (26%)	150 (29%)
Missing	55	8	8

PTE = Potentially traumatic experiences, CAMHS = Child and adolescent mental health services

^1^n (%), Reference group: class 1

### CAMHS Contact and Latent PTE Classes

The Situational trauma class and Interpersonal trauma class had a significantly higher odds ratio (OR) of contact with CAMHS than the low trauma class. Significant differences between classes persisted also after adjusting for sex and parents’ highest level of education. Compared to the Low trauma class, the adjusted odds ratio (AOR) of being in contact with CAMHS was 2.27 (95% CI [1.78, 2.87]), for the Situational trauma class and 3.26 (95% CI [2.61, 4.04]) for the Interpersonal trauma group (see Table 3). The Interpersonal Class further had a significantly higher odds ratio of being in contact with CAMHS compared to the Situational trauma class 1.43 (95% CI [1.06, 1.94]).

**Table 3:** Classes of Potentially Traumatic Experiences (PTEs) as CAMHS contact

	Model 1 (crude)			Model 2 (sex)			Model 3 (sex + education)		
Predictor	OR	95% CI	p-value	OR	95% CI	p-value	OR	95% CI	p-value
Class									
1	Ref			Ref			Ref		
2	2.34	1.84, 2.95	< 0.001	2.25	1.77, 2.84	< 0.001	2.27	1.78, 2.87	< 0.001
3	3.48	2.81, 4.30	< 0.001	3.32	2.67, 4.11	< 0.001	3.26	2.61, 4.04	< 0.001
2 vs. 3	1.49	1.11, 2.01	0.009	1.47	1.10, 1.99	0.011	1.43	1.06, 1.94	0.019

OR = Odds Ratio, CI = Confidence Interval, Ref = Reference group (Class 1), CAMHS = Child and adolescents mental health services

Model 1: Crude association. Model 2 is adjusted by sex. Model 3 is adjusted by sex and parental education

### Diagnosis and PTE latent classes

**Fig. 2:**
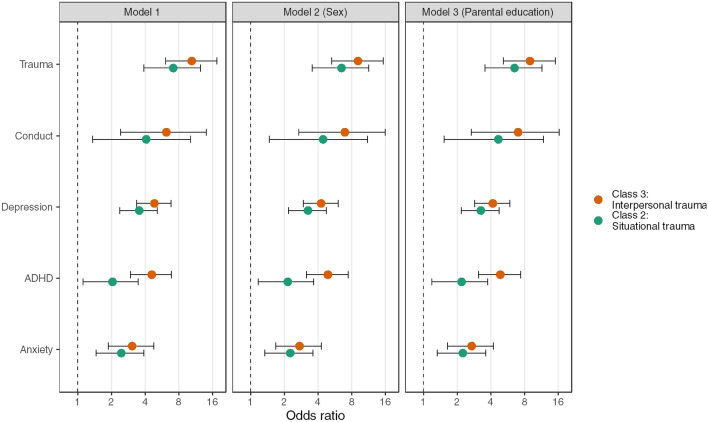
Regression coefficient plot of the associations between classes of potentially traumatic experiences (PTEs) and psychiatric diagnosis. Note. This figure is a visualization of the regression estimates from the logistic regression models examining the association between classes of potentially traumatic experiences (PTEs) and psychiatric diagnosis. The vertical dotted line represent the reference group (Class 1). The dots represent the point estimates, and the error bars represent 95% confidence intervals of the point estimates. The estimates are sorted in descending order based on the magnitude of the associations. The x-axis is log-transformed. Model 1 = crude, model 2 = adjusted by sex, model 3 = adjusted by sex and parental education

The Situational trauma class had significantly higher odds of receiving each of the ascertained diagnostic categories than the low trauma class in crude and adjusted analyses (Table 4 and Fig. 2). Adjusted odds of receiving a diagnosis of depression, anxiety, ADHD, conduct disorder, and trauma-related disorder were 3.25 (95% CI [2.17, 4.74])-, 2.25 (95% CI [1.33, 3.60])-, 2.19 (95% CI [1.19, 3.73])-, 4.66 (95% CI [1.53, 11.8])-, and 6.50 (95% CI [3.54, 11.5])-fold higher.

**Table 4:** Associations between classes of Potentially Traumatic Experiences (PTEs) and Psychiatric Diagnosis

		Model 1 (crude)			Model 2 (sex)			Model 3 (sex + education)		
Diagnosis	Predictor	OR	95% CI	p-value	OR	95% CI	p-value	OR	95% CI	p-value
Depression	Class									
	1	Ref			Ref			Ref		
	2	3.54	2.38, 5.13	< 0.001	3.25	2.18, 4.73	< 0.001	3.25	2.17, 4.74	< 0.001
	3	4.84	3.37, 6.82	< 0.001	4.26	2.96, 6.03	< 0.001	4.15	2.87, 5.90	< 0.001
	2 vs. 3	1.37	0.86, 2.20	0.193	1.31	0.82, 2.11	0.262	1.28	0.79, 2.06	0.314
Anxiety	Class									
	1	Ref			Ref			Ref		
	2	2.45	1.45, 3.90	< 0.001	2.26	1.34, 3.61	0.001	2.25	1.33, 3.60	0.001
	3	3.06	1.88, 4.77	< 0.001	2.73	1.67, 4.27	< 0.001	2.69	1.64, 4.22	< 0.001
	2 vs. 3	1.25	0.68, 2.37	0.485	1.21	0.64, 2.29	0.552	1.20	0.64, 2.28	0.573
ADHD	Class									
	1	Ref			Ref			Ref		
	2	2.05	1.11, 3.47	0.013	2.15	1.17, 3.65	0.008	2.19	1.19, 3.73	0.007
	3	4.58	2.96, 6.88	< 0.001	4.90	3.15, 7.40	< 0.001	4.85	3.11, 7.36	< 0.001
	2 vs. 3	2.23	1.19, 4.42	0.015	2.28	1.21, 4.51	0.013	2.22	1.17, 4.40	0.017
Conduct	Class									
	1	Ref			Ref			Ref		
	2	4.09	1.36, 10.2	0.005	4.44	1.47, 11.1	0.003	4.66	1.53, 11.8	0.003
	3	6.19	2.42, 14.1	< 0.001	6.95	2.69, 16.0	< 0.001	6.99	2.68, 16.3	< 0.001
	2 vs. 3	1.51	0.48, 5.15	0.483	1.57	0.50, 5.34	0.448	1.50	0.47, 5.14	0.494
Trauma-related disorders	Class									
	1	Ref			Ref			Ref		
	2	7.13	3.90, 12.5	< 0.001	6.48	3.54, 11.4	< 0.001	6.50	3.54, 11.5	< 0.001
	3	10.4	6.08, 17.5	< 0.001	9.10	5.28, 15.3	< 0.001	8.94	5.18, 15.1	< 0.001
	2 vs. 3	1.46	0.77, 2.82	0.246	1.40	0.74, 2.71	0.301	1.37	0.72, 2.66	0.335

OR = Odds Ratio, CI = Confidence Interval. Ref = Reference group (Class 1)

Model 1: Crude association. Model 2 is adjusted by sex. Model 3 is adjusted by sex and parental education

Also, the Interpersonal trauma class had significantly higher adjusted odds of receiving a diagnosis of depression, anxiety, ADHD, conduct disorder, and trauma-related disorder were 4.15 (95% CI [2.87, 5.90])-, 2.69 (95% CI [1.64, 4.22])-, 4.85 (95% CI [3.11, 7.36])-, 6.99 (95% CI [2.68, 16.3])-and 8.94 (95% CI [5.18, 15.1])-fold higher than in the low exposure class.

Pairwise comparisons between the Interpersonal and the Situational trauma class revealed a significantly higher OR of ADHD diagnosis in the Interpersonal trauma class (AOR = 2.22 (95% CI [1.17, 4.40]), p < 0.017). No other significant pairwise differences between the two classes were detected. All estimates from the logistic regression analyses are visually presented in Fig. 2, sorted by the magnitude of the associations.

## Discussion

This population-based study linked to national registry data revealed that adolescents can be grouped together to form three PTE classes, consisting of distinct patterns of trauma exposure: low trauma; Situational trauma; and Interpersonal trauma. Adolescents within the Situational trauma class and the Interpersonal trauma class had higher odds of being in contact with CAMHS compared to the Low trauma class. Compared to the Low trauma class, the Situational trauma class and Interpersonal trauma class had a significantly higher odds of all diagnostic categories, after adjusting for parental education and sex. There were, however, even more participants diagnosed with ADHD within the Interpersonal trauma class than the Situational trauma class.

Our study supports previous findings that there are underlying subgroups of adolescents that can be characterized by their distinct patterns of trauma exposure. In particular, our findings support a three-class model commensurate with three class models proposed by Brown et al. [6] and Curran et al. [15]. The types of experiences defining the classes in our study differed from the ones in Brown et al. [6], but were similar to the classes delineated by Curran et al. [15]. Both our study and Curran et al. [15] included a Low trauma class and a Situational trauma class. The Low trauma class consisted of 88% of the participants in our study and 85% in Curran et al. [15]. The Situational trauma class consisted of 6% of the participants in our study and 9% in the Curran et al. [15] study. The third class in our study was called Interpersonal trauma class, while in the Curran et al. [15] the third class was named the multi-victimization class. Nevertheless, both classes referred to Interpersonal PTEs, and both consisted of 6% of the participants. However, a difference between the studies was that in Curran et al. [15] the third class was characterized by exposure to all types of Interpersonal PTEs, while in our study, the class was characterized by increased exposure to direct or witnessed violence but lower exposure to death of a friend. Another difference was that the study by Curran et al. [15] was retrospective, with adults reporting on their childhood experiences, which could entail recall bias. Nevertheless, their findings were similar to our study, even though our was based on the reporting of adolescents during their adolescent period, which strengthens the findings by Curran et al. [15].

To our knowledge, our study is the first to examine the association between PTE classes, and CAMHS contact and psychiatric diagnoses. Our finding that both trauma classes were more likely to have contact with CAMHS than the low-trauma class supports previous finding that adolescents within CAMHS have more PTE exposure than those not in contact with CAMHS [1, 43]. The odds of being in contact with CAMHS were also markedly higher for the Interpersonal trauma class compared to the Situational trauma class. Even if there are no directly comparable studies, previous findings have revealed that Interpersonal trauma is more detrimental to mental health and cognitive function than Situational trauma [27, 35].

Being in either the Situational or Interpersonal trauma classes were both associated with higher odds of all studied diagnostic categories within CAMHS, even after adjusting for sex and parental education. Numerically, odds ratio for all diagnoses were higher in the Interpersonal trauma group compared to the Situational trauma group. However, risk was only significantly greater in the Interpersonal trauma class for ADHD.

The relationship between early life stress and ADHD is complex. Numerous studies have found that childhood maltreatment, a major form of Interpersonal trauma, is associated with an increased risk for ADHD [8], [17, 18, 22, 23], or can hasten the onset of ADHD [13], Rafael A. [17, 18]. Studies have also shown that children with ADHD are at increased risk for physical and emotional abuse by parents and peers [10, 13] as well as sexual victimization [11, 13]. Of note is a study by [50] in which they used a Mendelian randomization strategy to test for causal relationships between maltreatment and mental and physical health conditions. They reported a potential bidirectional causal relationship with maltreatment increasing the risk for ADHD and ADHD increasing the risk for subsequent maltreatment.

To further complicate matters, childhood trauma can lead to problems with affect regulation, behaviour regulation, stress tolerance, mood lability, anxiety, depression, and aggression [32, 46]. These symptoms can manifest themselves as disorganized affect, disruptive behaviours and distractibility, which all occur within ADHD [45]. Numerous studies have shown that exposure to Interpersonal trauma during childhood can chronically and pervasively alter social, psychological, and cognitive development [9]. Consequently, there is a possibility that clinicians may mistake such symptoms for those of ADHD, without necessarily discerning a history of Interpersonal trauma, which may be more difficult to detect than Situational trauma [28], [45]. In addition, early deprivation [33] and harsh corporal punishment [40]; [47] are associated with brain changes that overlap with reported brain changes in youths with ADHD. Finally, pre-peri-and posttraumatic risk factors other than PTE exposure in itself, might impact some of the variation in the association between PTEs and different diagnoses [12].

### Strengths and Limitations of the Study

The strength of our study is the measure of both PTEs and psychopathology within the developmental stage of childhood and adolescence. In addition, it consisted of survey information and national registry data from CAMHS from many participants, which provided us with a unique possibility of comparing PTE classes in a clinical vs a non-clinical group. Diagnoses were set by professional practicing clinicians within CAMHS, and we had information about all diagnostic categories. By using a person-centered approach when examining clusters of PTEs our study managed to identify homogenous subgroups while still recognizing the heterogeneity of PTEs experienced at a population level. Our use of latent class analysis as a statistical measure of PTEs in adolescence provided further insight into the co-occurrence of PTEs and gave a unique way of comparing patterns of PTEs across the diagnostic spectrum.

Several limitations must nevertheless be considered when interpreting the findings of the current study. Even if we know that most of the sample had been in contact with CAMHS before the onset of the youth@hordaland survey, a primary limitation is that we have not included when the PTE was experienced in relation to registration in CAMHS. Hence, we are therefore unable to determine the temporal order between the variables that we have included in our analyses. Participants with higher levels of PTE exposure may be more vulnerable to the development of different psychopathology or those with psychopathology may be at a higher risk of experiencing PTEs [12, 31]. Also, our study examined cumulative PTE exposure only rather than specific associations between PTE types and diagnoses, which entails an assumption that categories of adversity are of equal weight that might not necessarily be the case and could negatively impact the external validity of our findings.

Our PTE categories are limited to ten categories. Previous studies vary in terms of number of PTEs and type of PTEs included, which could impact findings [15, 35]. Even if our study included the most common PTEs, a possible limitation is not including medical trauma and exposure to war. We could argue that these PTEs could be placed within our category “a catastrophe or serious accident”. However, the lack of specificity could impact how many participants identify with the category which could have led to underreporting of these experiences. It is also a limitation that we have not included bullying, and that acts of violence were limited to exposure from an adult only. The low occurrence of certain diagnosis in the sample impacts the reliability of the point estimates, which thus should be interpreted with some caution.

Although LCA is a powerful statistical procedure, it has limitations. Exact class assignment is not guaranteed. LCA assigns individuals to classes based on their probability of being in classes given the pattern of scores they have on indicator variables. The exact number or percentage of sample members within each class cannot be determined, seeing that class assignment is based on probabilities. A complicating factor in LCA in general and in our study is the subjective naming of the assigned classes. The complexity of the classes may not adequately be reflected in the chosen name of the class.

### Implications for Future Research

There is a need for additional research that can expand our knowledge of the co-occurrence of PTEs and psychopathology across the diagnostic spectrum. Such studies are encouraged to replicate our study design but should include an even greater variety of PTEs, with a possible outcome of more PTE latent classes. There is a need for well-designed longitudinal studies that better can establish the temporal order of PTE exposure and diagnoses, limiting the sample to the child and adolescent population only. By providing findings on the temporal order, this might give further insight into the question of trauma as a risk factor for psychopathology or psychopathology as a risk factor for PTEs, or bidirectional.

## Summary

The results from the current study supported that PTEs co-occur and may form interrelated patterns of exposure. The patterns were predictive of contact with child and adolescent mental health services (CAMHS) and of being diagnosed with a psychiatric disorder in CAMHS. More adolescents were diagnosed with ADHD within the Interpersonal trauma class. The findings suggest a need to pay attention to adolescents being exposed to both patterns of traumas, but in particular, adolescents exposed to Interpersonal trauma. Key takeaway message for clinicians when detecting PTE exposure in adolescents, is to systematically screen for additional PTEs and pay attention to patterns of exposure, in addition to assessing mental health problems across the diagnostic spectrum. Patterns of exposure could be an indication of adolescents´ level of vulnerability and gravity of mental health problems after being exposed to PTEs.
